# Comparison of human macrophages derived from peripheral blood and bone marrow

**DOI:** 10.1093/jimmun/vkae032

**Published:** 2025-03-05

**Authors:** Hannah L Smith, Russell B Foxall, Patrick J Duriez, Emma L Teal, Adam D Hoppe, Janos M Kanczler, Juliet C Gray, Stephen A Beers

**Affiliations:** Antibody and Vaccine Group, Centre for Cancer Immunology, School of Cancer Sciences, Faculty of Medicine, University of Southampton, Southampton, United Kingdom; Bone and Joint Research Group, Human Development and Health, Institute of Developmental Sciences, Faculty of Medicine, University of Southampton, Southampton, United Kingdom; Antibody and Vaccine Group, Centre for Cancer Immunology, School of Cancer Sciences, Faculty of Medicine, University of Southampton, Southampton, United Kingdom; Antibody and Vaccine Group, Centre for Cancer Immunology, School of Cancer Sciences, Faculty of Medicine, University of Southampton, Southampton, United Kingdom; Antibody and Vaccine Group, Centre for Cancer Immunology, School of Cancer Sciences, Faculty of Medicine, University of Southampton, Southampton, United Kingdom; Department of Chemistry, Biochemistry and Physics, South Dakota State University, Brookings, South Dakota, United States; Bone and Joint Research Group, Human Development and Health, Institute of Developmental Sciences, Faculty of Medicine, University of Southampton, Southampton, United Kingdom; Antibody and Vaccine Group, Centre for Cancer Immunology, School of Cancer Sciences, Faculty of Medicine, University of Southampton, Southampton, United Kingdom; Antibody and Vaccine Group, Centre for Cancer Immunology, School of Cancer Sciences, Faculty of Medicine, University of Southampton, Southampton, United Kingdom

**Keywords:** ADCP, bone marrow, macrophages, PBMCs, phagocytosis

## Abstract

Macrophage differentiation, phenotype, and function have been assessed extensively in vitro by predominantly deriving human macrophages from peripheral blood. It is accepted that there are differences between macrophages isolated from different human tissues; however, the importance of anatomical source for in vitro differentiation and characterization is less clear. Here, phenotype and function were evaluated between human macrophages derived from bone marrow or peripheral blood. Macrophages were differentiated by adherence of heterogenous cell populations or CD14 isolation and polarized with IFNγ and LPS or IL-4 and IL-13 for 48 hours before evaluation of phenotype and phagocytic capacity. The presence of stromal cells in bone marrow heterogenous cultures resulted in a reduction in macrophage purity compared to peripheral blood, which was negated after CD14 isolation. Phenotypically, monocyte-derived macrophages (MDMs) derived from peripheral blood and bone marrow resulted in similar expression of classical and polarized macrophages markers, including CD14, HLA-DR, CD38, and CD40 (increased after IFNγ/LPS), and CD11b and CD206 (elevated after IL-4/IL-13). Functionally, these cells also showed similar levels of Fc-independent and Fc-dependent phagocytosis, although there was a nonsignificant reduction of Fc-dependent phagocytosis in the bone marrow derived macrophages after IFNγ/LPS stimulation. In summary, we have identified that human MDMs differentiated from peripheral blood and bone marrow showed similar characteristics and functionality, suggesting that isolating cells from different anatomical niches does not affect macrophage differentiation after CD14 isolation. Consequently, due to high yield and ready availability peripheral blood derived macrophages are still the most suitable source.

## Introduction

Macrophages are important innate immune cells that are involved in the detection and destruction of pathogens, with key roles in immune regulation and tissue homeostasis. Macrophages are derived either from monocyte precursors^1^ or are established during embryonic development and maintained by local proliferation independent of circulating monocytes.^2^^,^^3^ While both monocyte-derived and tissue-resident macrophages have distinctive roles in immune response and homeostasis, evidence has shown both types of macrophages can have their function reprogrammed to adapt to the specific environmental needs; this includes monocyte-derived macrophages acting as tissue-resident macrophages and showing the ability to self-maintain.^4^^,^^5^

Understanding macrophage function, particularly their immune modulatory capacity, has been an area of intense focus for many years as this could be exploited for the development of new treatments for cancer and autoimmune diseases. Macrophages have been broadly classified by their wide spectrum of polarization (linked phenotypic and functional) states^6^ extending from proinflammatory (M1-like) macrophages,^7^ to alternatively activated (M2-like) macrophages which stimulate proliferation and tissue repair.^8^ In several solid tumors macrophage polarization has been correlated with prognosis, indicating a reduced survival rate linked with a higher number of M2-like macrophages in the tumor microenvironment.^9^^,^^10^ Similarly, infiltration of monocytes and macrophages has been found in several autoimmune diseases, including rheumatoid arthritis and inflammatory bowel disease, where they show a range of polarization phenotypes depending on the stage of disease and microenvironment.^11^

The significance of macrophage polarization in a range of diseases highlights the importance of understanding the interactions and functions of these cells in vitro, to investigate how these cells differentiate for the development and testing of new therapies. Primary human macrophages studied in vitro are generally isolated and differentiated from PBMCs,^12^^,^^13^ where they have been used to investigate a variety of macrophage roles, including within the tumor microenvironment.^14^^,^^15^ This choice of tissue source simply relates to its ready availability, ease of access, and volume of material obtainable, but does not mean peripheral blood monocyte derived macrophages (pMDMs) are necessarily the most robust and representative source of macrophages for in vitro analysis relating to different anatomical contexts. Indeed, due to differences in tissue availability and abundance, murine macrophage assays are often derived from the bone marrow.

The similarity between in vitro differentiated blood and bone-derived macrophages in humans is not well studied. The majority of those reports that can be identified were performed in the 1970s and 1980s using markedly different protocols to current optimized methods, notably recombinant M-CSF was not available at that time.^16–18^ More recent studies have focused on specific diseases, for example how the replication of the hepatitis E virus compared in monocyte-derived and bone marrow–derived macrophages,^19^ not on the functional differences of the macrophages themselves. A study by van Leeuwen-Kerkhoff et al.^20^ has identified phenotypic and function differences between dendritic cells in the peripheral blood and bone marrow, which could suggest possible variations in other immune cell populations, including macrophages.

Here, phenotypic differences between macrophages differentiated from PBMCs (pMDMs), and bone marrow (bMDMs) were compared from both heterogenous populations and after CD14 isolation. These analyses identified minimal differences after polarization into M1 and M2-like macrophages. Functionally, phagocytosis, both Fc dependent and independent, resulted in a nonsignificant trend for a reduction in the level of phagocytosis in M1-like bMDMs compared to pMDMs, but no change in M0 and M2-like phagocytosis.

## Materials and methods

### Primary samples

For all tissue used, informed patient consent was obtained in alignment with the Declaration of Helsinki. Ethical approval was obtained for using healthy donor leukocyte cones from the NHS blood and transplant service (REC number 16/ES/0048), and peripheral blood samples from chronic lymphoblastic leukemia (CLL) patients (REC number 10/H0504/187). Femoral head and bone marrow was obtained from patients undergoing elective hip replacement surgery at the University Hospital Southampton NHS Foundation Trust and Spire Southampton Hospital (REC number 18/NM/0231).

### Isolation of immune cells

PBMCs were isolated from healthy donor leukocyte cones by density gradient centrifugation at 800 × *g* for 20 minutes (Lymphoprep, FisherScientific 11508545) and contaminating platelets eliminated by 3 slow-speed centrifugation washes in PBS/EDTA (PBS + 2 mM EDTA) at 150 × *g*, 15 minutes. Cells were isolated from bone fragments through vigorous shaking in PBS/EDTA, then washed in PBS/EDTA (300 × *g*, 5 minutes). The red blood cells were then lysed (1 L PBS + 8.4 g ammonium chloride + 1 g potassium hydrogen carbonate) for 5 minutes and resuspended in alpha MEM + 1% P/S (100 U/ml penicillin + 100 ug/ml streptomycin).

### Macrophage differentiation

Isolated PBMCs and bone marrow cells were plated at a concentration between 1 to 2 × 10^7^ cells/ml in αMEM + 1% P/S + 1% human AB serum (Sigma, H3667) and differentiated into macrophages as previously described.^15^ Briefly, the cells were incubated for 2 hours before nonadherent cells were removed through repeated washes in PBS. The cells were then incubated overnight in complete alpha MEM media (alpha MEM + 1% P/S + 10% FCS), where 100 ng/ml of M-CSF (made in-house using published sequences) was then added, and the macrophages were differentiated for 7 days. PBMC and bone marrow samples were also used for the isolation of CD14 positive cells prior to differentiation using either the Miltenyi CD14 MicroBeads Isolation kit (130-050-201), or StemCell EasySep CD14+ selection kit (17858), both performed according to manufacturer’s instructions. The resulting monocytes (containing both classical and nonclassical) were cultured at 1 × 10^6^ cells/ml in complete alpha MEM + M-CSF for 7 days. On day 7 the macrophages were either analyzed by flow cytometry or polarized with 2 ng/ml IFNγ (PeproTech, 300-02) and 50 ng/ml LPS (Sigma, L3024) for M1, or 10 ng/ml IL-4 (PeproTech, 200-04) and 10 ng/ml IL-13 (PeproTech, 200-13) for M2, as previously described.^15^

### Flow cytometry

Cells were harvested by gentle scraping after 15-minutes incubation in PBS on ice, then stained for 30 minutes at 4 °C in the dark, with a panel of antibodies to cell surface markers (Table 1). After incubation the cells were centrifuged and resuspended in FACS buffer (1 × PBS + 5 µg/ml (w/v) BSA + 0.1% (v/v) Azide) before being analyzed by flow cytometry (FACS Canto II, Becton Dickinson), and further analyzed using FlowJo Version 10 software (FlowJo LLC). A representative flow cytometry gating strategy is provided in Fig. S1.

**Table 1.** Antibodies for flow cytometry.

Antibody	Fluorochrome	Isotype	Company
HLA-DR	PerCp-Cy5.5	Mouse IgG2a	Biolegend, 307629
CD14	APC	Mouse IgG1	Biolegend, 367117
CD11b	Pacific Blue	Rat IgG2b	Biolegend, 101224
CD38	PE	Mouse IgG1	Biolegend, 356604
CD40	APC-Cy7	Mouse IgG1	Biolegend, 334324
CD206	PE	Mouse IgG1	Biolegend, 321106

### Phagocytosis

To assess Fc-independent phagocytosis 1.2 × 10^6^ 3-µm BSA-coated beads were incubated with the macrophages for 1 hour before being analyzed by flow cytometry. As previously reported,^21^ 3 µm beads (Polysciences, 17134-15) were labelled with AF488 BSA prior to use before being analyzed by flow cytometry. Antibody dependent cellular phagocytosis (ADCP) was assessed as reported previously.^15^ CLL cells were used as target cells, stained with CFSE (ThermoFisher C34554) and opsonized with 10 μg/ml of rituximab hIgG1 antibody or trastuzumab as an isotype control prior to ADCP (antibodies gifted by the Oncology Pharmacy at Southampton General Hospital). The phagocytic index of the macrophages was calculated by deducting the percentage ADCP of the isotype control from the opsonized cells before normalizing this to the M0 cells from the same donor.

### Statistics

Experimental data were analyzed using GraphPad Prism version 10 software. Results were expressed as mean ± SD. Significance was assessed using either a one-way ANOVA with Tukey’s post hoc test (>2 groups), or an unpaired T test (<2 groups). The statistical test used is stated on each figure. Values of *P* ≤ 0.05 were considered significant. Significance presented as *<0.05, **<0.01, ***<0.001, ****<0.0001.

## Results

### Macrophage differentiation from heterogenous cell populations

**Figure 1.**
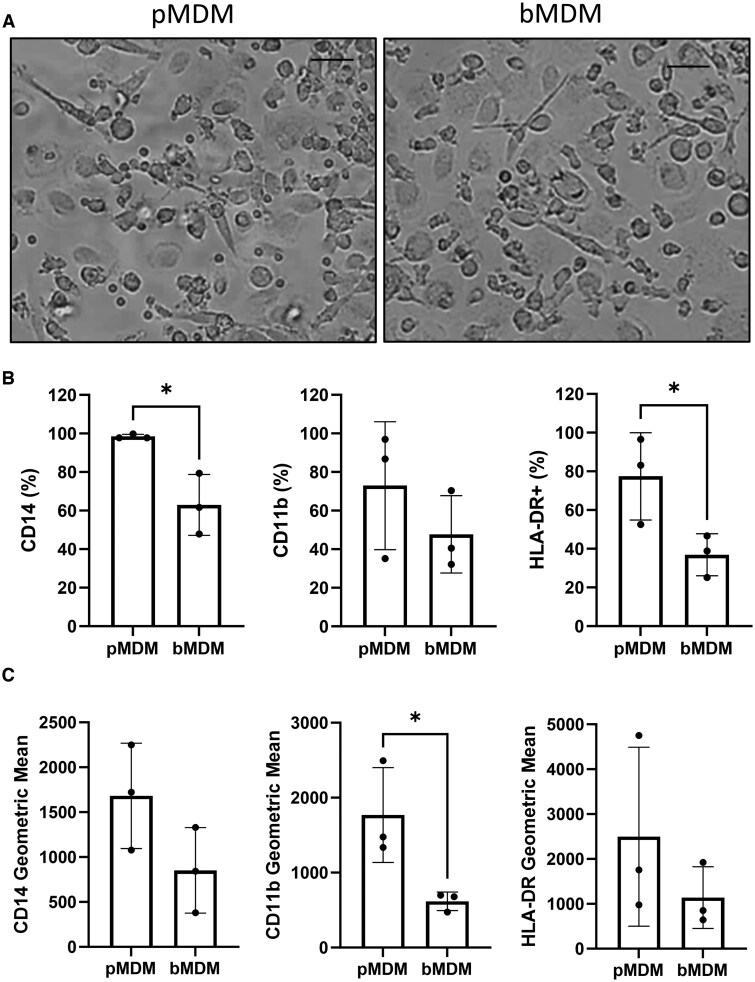
Phenotype of macrophages derived from heterogenous cell populations. (A) Representative images of pMDMs and bMDMs. Adherent cells were isolated and differentiated with M-CSF for 7 days. Scale bar = 100 μm. (B) The percentage of pMDMs and bMDMs expressing the macrophage markers CD14, CD11b, and HLA-DR. (C) The geometric mean of pMDMs and bMDMs expressing CD14, CD11b, and HLA-DR. N = 3 samples with data points representing the mean of 3 technical replicates. Results presented as mean ± SD; statistics analyzed using an unpaired T test; significance presented as *<0.05.

To assess whether there were any phenotypic differences between macrophages derived from blood and bone, pMDM and bMDMs were first differentiated from heterogenous source populations. Adherent cells from PBMC or bone marrow suspensions were cultured for 7 days with M-CSF before being analyzed by flow cytometry. Three markers were used to characterize the macrophages: CD14, a monocyte/macrophage differentiation marker,^22^ CD11b, an integrin involved in adhesion and cell migration, which is highly expressed on macrophages,^23^ and HLA-DR which is responsible for antigen presentation and initiation of the inflammatory response.^24^ Both pMDM and bMDM populations showed similar morphologies with heterogenous populations of large and small, round and elongated cells; representative images were taken after 7 days of differentiation (Fig. 1A). However, flow cytometry analysis of these macrophages indicated phenotypic differences (flow cytometry gating strategy in Fig. S1). Figure 1B shows a reduction in the percentage of cells expressing all three markers assessed in the bMDMs, which was statistically significant for CD14 and HLA-DR. This trend was also observed in the geometric mean (Fig. 1C), which was statistically significant for CD11b. This suggests that the bMDM samples contained a lower number of macrophages compared to the pMDMs, and that those macrophages positive for these markers also expressed lower levels.

**Figure 2.**
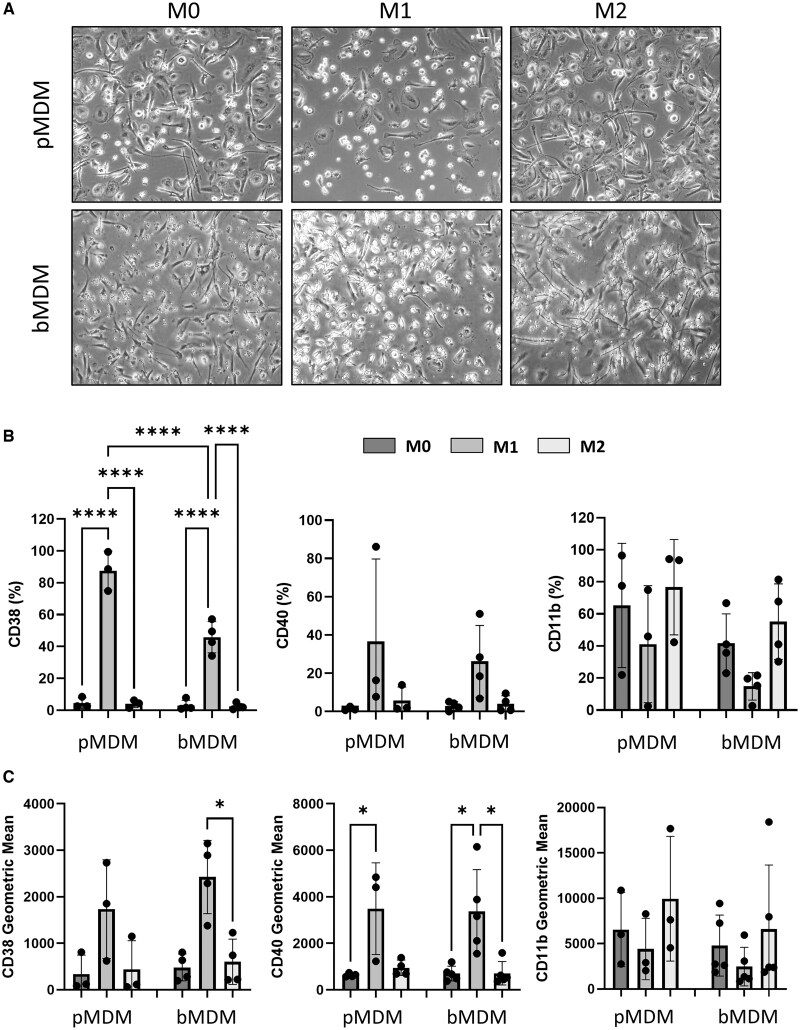
Phenotype of polarized macrophages derived from heterogenous cell populations. Adherent cells were isolated and differentiated with M-CSF for 7 days before being polarized for a further 48 hours, with either IFN-γ and LPS (M1), or IL-4 and IL-13 (M2). (A) Representative images of M0, M1, and M2-polarized pMDMs and bMDMs. Scale bar = 100 μm. (B) The percentage of polarized pMDMs and bMDMs expressing M1 markers CD38 and CD40, and an M2 marker CD11b. (C) The geometric mean of polarized pMDM and bMDM cells expressing CD38, CD40, and CD11b. N = 3–4 samples with each data point representing the mean of 3 technical replicates. Results presented as mean ± SD; statistics analyzed using a one-way ANOVA; significance presented as *<0.05, ****<0.0001.

Macrophages have a variety of functions linked to their polarization state, extremes of which can be represented by M1 and M2-like phenotypes. To identify differences in response to polarization, macrophages were differentiated for 7 days with M-CSF, then polarized for a further 48 hours with IFNγ and LPS (M1) or IL-4 and IL-13 (M2) before being analyzed by flow cytometry. Representative images (Fig. 2A) show the pMDMs and bMDMs had a similar morphology across activation states, including heterogenous M0 macrophages, rounder M1-like macrophages, and more elongated M2-like macrophages. Figure 2B shows the expression of polarization markers including CD38 and CD40, which are established M1-like markers,^25^^,^^26^ and CD11b, which has been identified as an M2-like marker.^27^^,^^28^ These results demonstrate similar patterns of expression between the polarized macrophages, with an increase in CD38 and CD40 for the M1-like macrophages and an increase in CD11b in M2-like. However, the bMDMs had lower expression of these markers compared to the pMDMs, which was significant for CD38. In contrast, the geometric means of the three macrophage markers were similar between pMDM and bMDM, suggesting that once polarized, although there were fewer cells expressing these markers, those that were positive had similar expression levels.

The bMDMs displayed a reduction in the expression of various macrophages markers compared to pMDMs, both after initial differentiation (7 days) and subsequently after polarization (+48 hours). It was notable, that the cell yield was considerably lower in the bone marrow samples compared to peripheral blood, with on average 3–4 times fewer cells and larger donor variation. Double the number of bone marrow cells also needed to be plated initially to result in similar confluency to pMDM after 7 days, with more cellular debris and lipid residue also found in the bMDM wells. Furthermore, in the majority of bMDM cultures adherent bone marrow stromal cells were also identified (Fig. S2). This suggested that the bMDM cultures were contaminated by stromal cells, which resulted in an overall decrease in the number of differentiated macrophages.

### Macrophage differentiation from CD14-isolated cells

**Figure 3.**
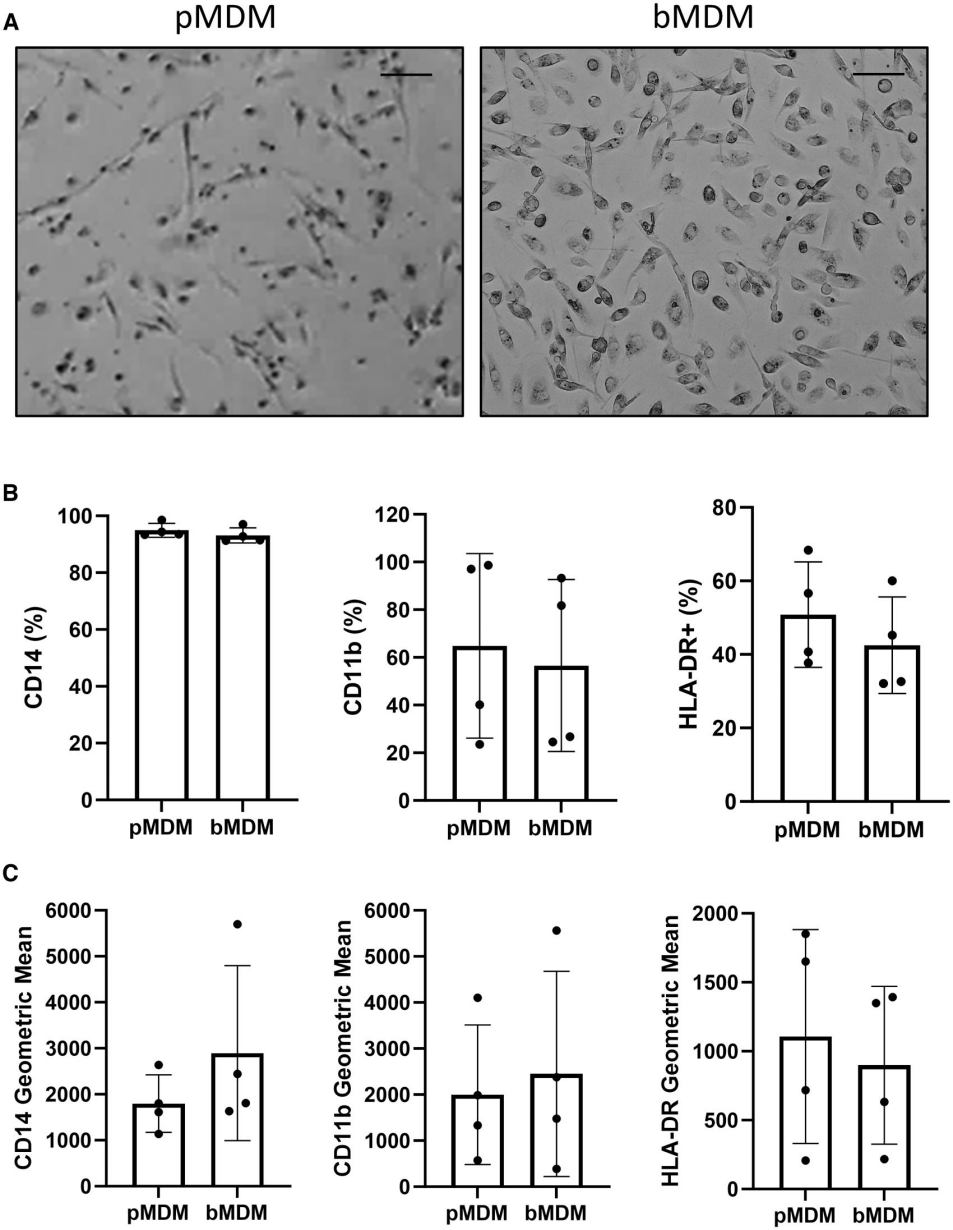
Macrophage phenotype after 7 days differentiation from CD14-isolated monocytes. CD14 cells from PBMCs or bone marrow suspensions were isolated using magnetic cell sorting, then differentiated with M-CSF for 7 days. (A) Representative images of pMDM- and bMDM-differentiated cells. Scale bar = 100 μm. (B) The percentage of pMDMs and bMDMs expressing macrophage markers CD14, CD11b, and HLA-DR. (C) The geometric mean of pMDM and bMDM cells expressing CD14, CD11b, and HLA-DR. N = 4 patients with data points representing the mean of 3 technical replicates. Results presented as mean ± SD; statistics analyzed using an unpaired T test; no significance determined.

To overcome the infiltration of stromal cells in the bMDM macrophage populations and their potential to impact on comparison to pMDM, CD14 positive cells were first isolated from both the heterogenous cell suspensions. These CD14 positive cells (similar average purity >90% obtained from both sources) were then incubated for 7 days with M-CSF, and their morphology and phenotype compared. The morphology of the CD14-isolated macrophages 7 days after differentiation were similar, with a mixture of both elongated and more-rounded cells (Fig. 3A). These cells also show a similar morphology to the heterogenous isolated macrophages (Fig. 1), although the CD14-isolated cells were more uniform and did not show areas of stromal cell contamination as previously identified in the bMDMs (Fig. S2). Notably, despite CD14 isolation there was still a small amount of lipid residue visible in the bMDMs at the time of plating and during washing steps. Although donor variability was evident with both sources, Fig. 3B demonstrates similar percentage expression for the 3 macrophage markers, CD14, CD11b, and HLA-DR, across sample types with a similar trend reflected in their geometric means (Fig. 3C). Similarities in expression were also observed for CD47 and SIRPα (Fig. S3A, B). This demonstrated that the isolation of CD14 positive cells was effective in removing previously contaminating stromal cells and that these contaminants were largely responsible for the differences previously observed.

**Figure 4.**
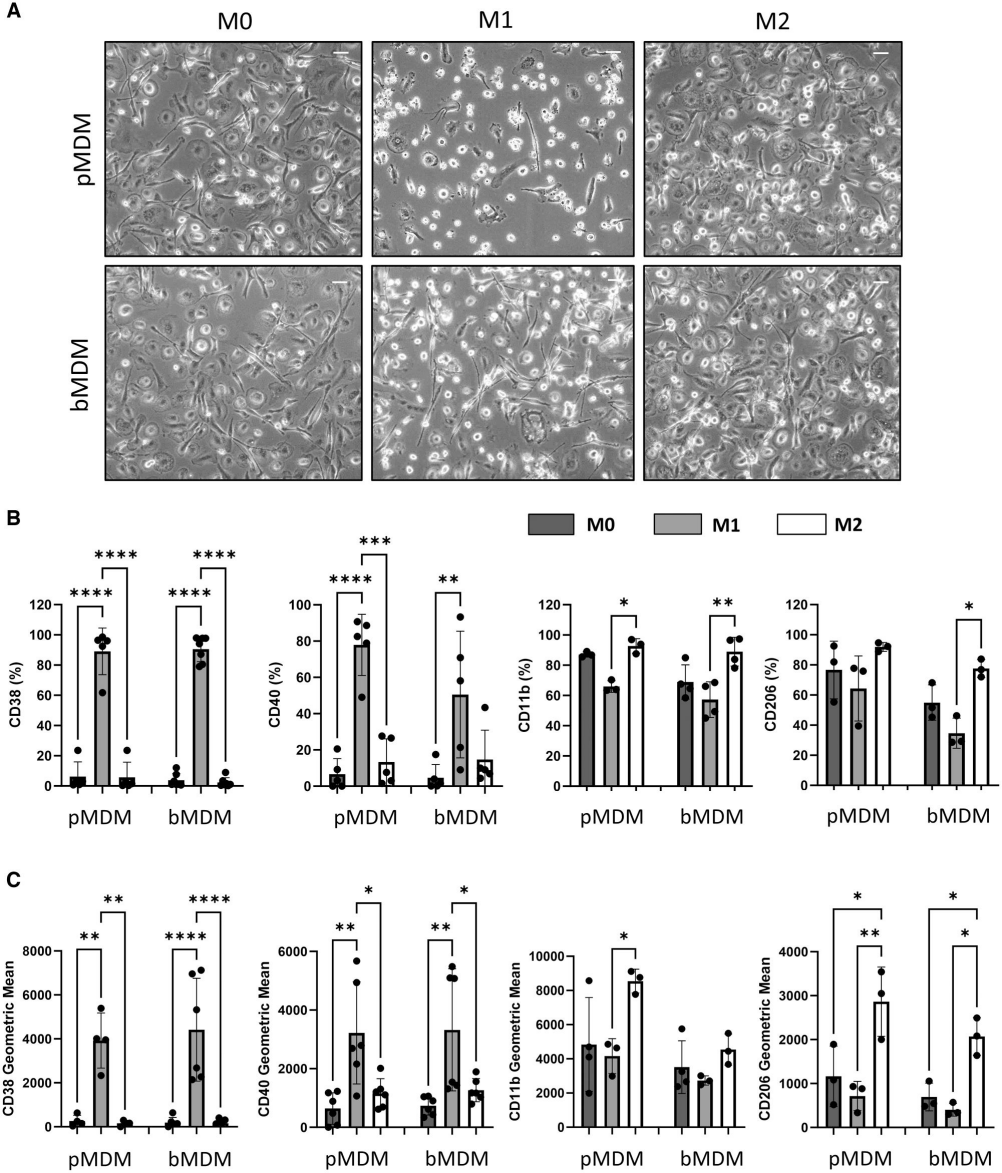
Phenotype of polarized macrophages differentiated from CD14-isolated monocytes. (A) Representative images of pMDMs and bMDMs polarized into M1 and M2-like macrophages. Cells were isolated from PBMC or bone marrow suspensions and differentiated with M-CSF for 7 days before being polarized for a further 48 hours. M1-like macrophages were incubated with IFN-γ and LPS; M2-like macrophages were incubated with IL-4 and IL-13. Scale bar = 100 μm. (B) The percentage of polarized pMDMs and bMDMs expressing M1 markers CD38 and CD40, and M2 markers CD11b and CD206. (C) The geometric mean of polarized pMDM and bMDM cells expressing CD38, CD40, CD11b, and CD206. N = 3–4 samples with each data point representing the mean of 3 technical replicates. Results presented as mean ± SD; statistics analyzed using a one-way ANOVA; significance presented as *<0.05, **<0.01, ***<0.001, ****<0.0001.

The CD14-isolated pMDMs and bMDMs were then polarized to compare the phenotypes of M1 and M2-like macrophages (Fig. 4). There were similarities in morphology between the cells derived from the PBMC and bone marrow (Fig. 4A), where M1-like macrophages consisted of larger, rounded cells, and the M2-like macrophages were straighter and more elongated. These macrophages also showed a similar morphology to the heterogenous isolated cells (Fig. 2), but again without the stromal cell contamination previously identified in bMDMs (Fig. S2). There was similar expression of CD38 (Fig. 4B) between the M1-polarized pMDMs and bMDMs, but the expression of CD40 was decreased in the bMDM populations compared to the pMDM, suggesting potential subtle differences in M1 phenotype. Assessment of the expression of the mannose receptor, CD206, was also used alongside CD11b to assess M2-like polarization.^29^ Both markers showed a similar level of expression, with only a slight reduction of CD206 in the bMDMs compared to pMDMs. There were also similar trends in the geometric mean of M1-like markers for the 2 sources (Fig. 4C) and in FcγR expression in all polarization conditions (Fig. S3C–F). In contrast, there was a reduction in the geometric means of M2-like markers in the bMDM samples compared to the pMDMs, although this was not significant. From this we inferred that although there were similar numbers of CD11b and CD206 positive macrophages after M2 polarization, there was a reduction in expression, suggesting a marginal reduction in M2 characteristics.

### Fc-independent phagocytosis

**Figure 5.**
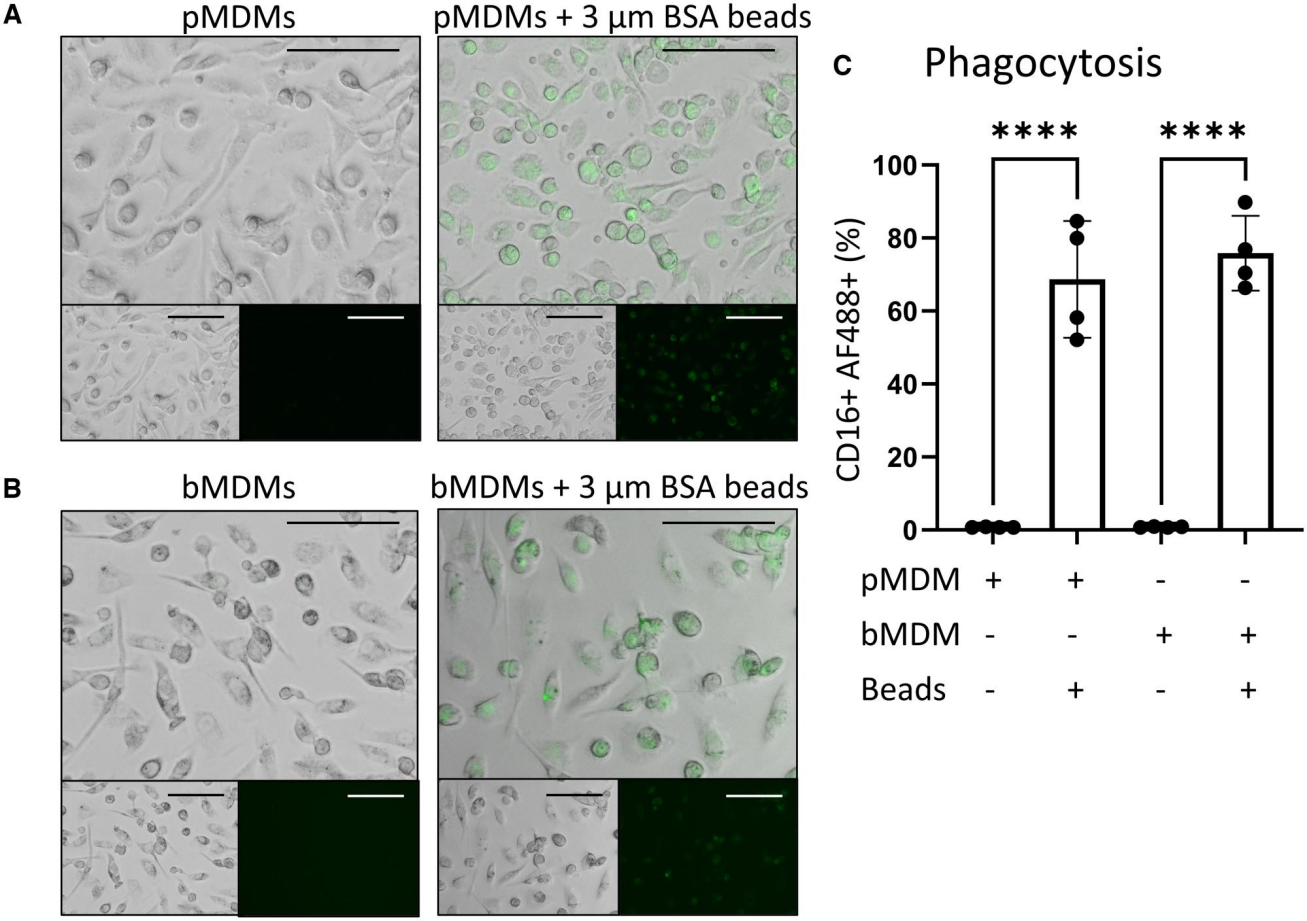
Fc-independent phagocytosis of CD14-isolated macrophages. CD14 positive cells were isolated using magnetic isolation from PBMCs or bone marrow suspensions, then incubated for 7 days with M-CSF. 3-μm BSA beads labelled with AF488 were then incubated with established macrophages for one hour. Uptake of the BSA beads (green) in (A) pMDMs and (B) bMDMs. Scale bar = 100 μm. (C) Percentage of AF488+ macrophages when incubated with and without the BSA beads. N = 4 samples with each data point representing the mean of 3 to 5 technical replicates. Results presented as mean ± SD; statistics analyzed using a one-way ANOVA; significance presented as ****<0.0001.

Overall, CD14-isolated M0 macrophages derived from PBMCs and bone marrow cells (pMDM and bMDM respectively) showed a similar phenotype after 7-days differentiation with M-CSF, but some nonsignificant trends were observed after M1 and M2-like polarization. To test whether these M0 pMDMs and bMDMs were similarly functionally active, they were assessed for their Fc-independent phagocytic potential. Previous reports have established that phagocytosis of beads larger than 15 μm is FcγR dependent;^21^^,^^30^ thus, 3-μm BSA coated beads fluorescently labelled with Alexa Fluor 488 were employed and cocultured with macrophages for 1 hour to compare their Fc-independent phagocytic uptake (Fig. 5). The presence of the labelled BSA beads (green) were identified within the pMDMs (Fig. 5A) and bMDMs (Fig. 5B) demonstrating phagocytosis had occurred. Figure 5C illustrates comparable percentage of phagocytosis between MDMs from the 2 sources, suggesting tissue source did not affect phagocytic function in in vitro–differentiated macrophages.

### Fc-dependent phagocytosis

**Figure 6.**
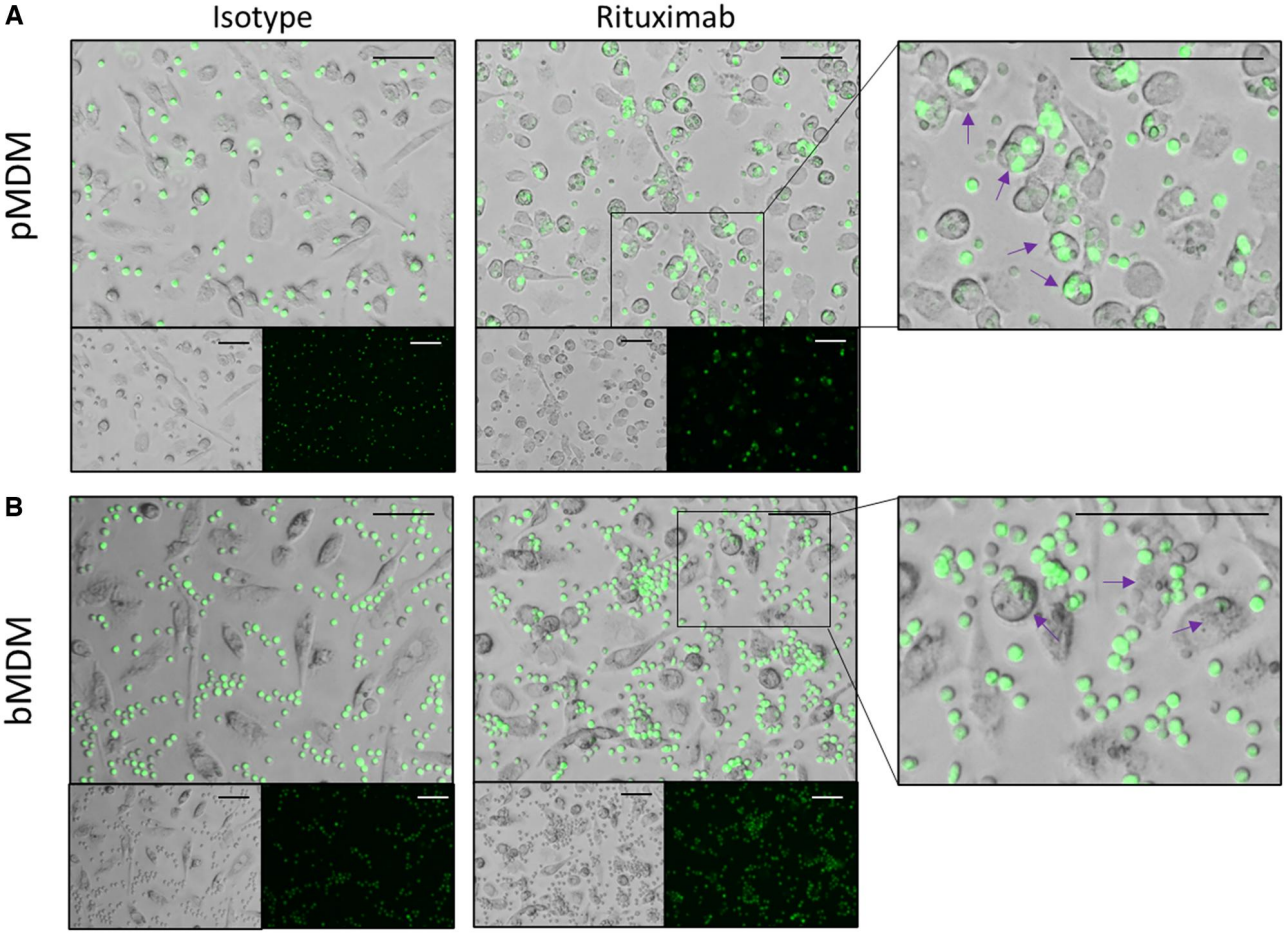
ADCP of macrophages differentiated from CD14-isolated monocytes. CD14 positive cells were isolated from PBMCs and human bone marrow suspensions and cultured for 7 days with M-CSF to generate pMDM and bMDM, respectively. CFSE-labelled CLL cells were then incubated with macrophages for 1 hour after being opsonized with rituximab or an isotype control. Uptake of CFSE+ CLL cells (green) by either (A) pMDMs or (B) bMDMs. Cells highlighted by purple arrows indicate macrophages that have engulfed CLL cells. Scale bar = 50 μm. Representative images from N = 5.

Macrophages were also assessed for ADCP. Here macrophages were polarized with either IFN-γ and LPS (M1) or IL-4 and IL-13 (M2), then cocultured for 1 hour with CFSE-labelled CLL cells that had been opsonized with either the anti-CD20 chimeric human IgG1 antibody, rituximab, or trastuzumab as an isotype control. Phagocytosis of rituximab opsonized CLL cells can be clearly observed in M0 pMDMs (Fig. 6A) and bMDMs (Fig. 6B) (examples indicated by purple arrows), compared to very low to no phagocytosis in controls.

**Figure 7.**
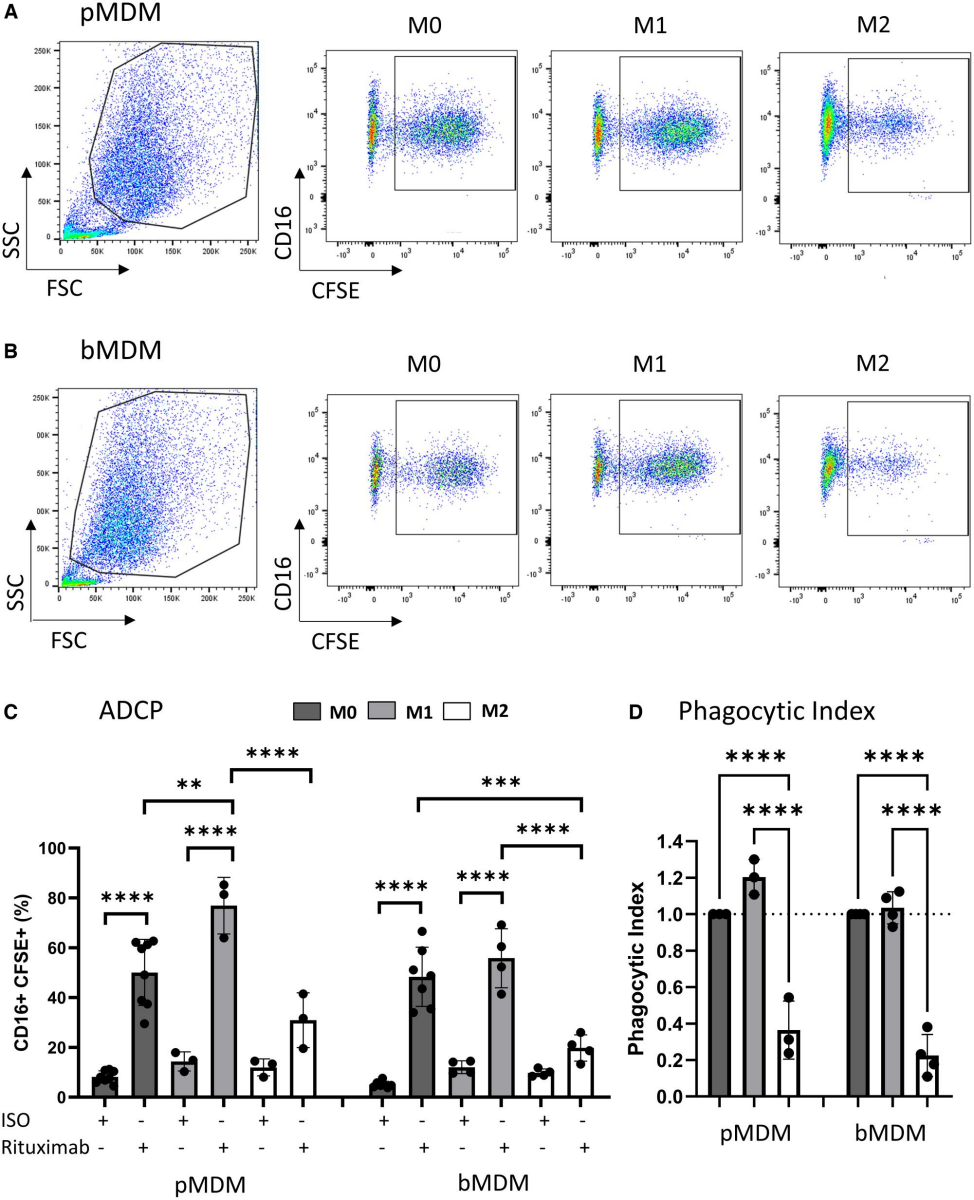
Flow cytometry analysis of ADCP from macrophages differentiated from CD14-isolated monocytes. CD14 positive cells were isolated from PBMCs or bone marrow suspensions and cultured for 7 days with M-CSF, then polarized for 48 hours with IFNγ and LPS (M1) or IL-4 and IL-13 (M2). CFSE-labelled CLL cells opsonized with either rituximab or an isotype control were then cocultured with the macrophages for 1 hour. The (A) pMDMs and (B) bMDMs were then stained for CD16 and assessed by flow cytometry identifying the CD16+CFSE+ cells. (C) Percentage uptake of CLL cells by M0-, M1-, and M2-polarized pMDMs and bMDMs. (D) Phagocytic index of polarized macrophages normalized to M0 cells. N = 3–8 samples with each data point representing the mean of 3 to 5 technical replicates. Results presented as mean ± SD; statistics analyzed using a one-way ANOVA; significance presented as **<0.01, ***<0.001, ****<0.0001.

Macrophages were further analyzed for phagocytic effector capacity using flow cytometry by comparing the percentage of CD16+CFSE+ cells (macrophages that had phagocytosed CLL cells). Clear differences can be seen between the ADCP of polarized cells, with M1-like having the highest percentage and M2-like the lowest (Fig. 7A and B), as observed in previous reports.^15^^,^^21^^,^^31^  Figure 7C summarizes the differences in ADCP between the pMDMs and bMDMs. For both, there is a clear decrease in ADCP with M2-like macrophages compared to M0, which showed similar levels with both cell sources. In contrast, M1-like bMDMs demonstrated similar percentages of phagocytosis to M0 and were lower compared to M1-like pMDMs. When these data were normalized to a phagocytic index (Fig. 7D), there was no difference between the phagocytic capacity of M1-like and M0 bMDMs, suggesting that bMDMs did not have the same capacity for M1 effector function as pMDMs. This difference in effector capacity was not observed in the previous characterization of these cells, which showed similarly higher levels of CD38 and CD40 expression in M1-like macrophages compared to M0 (Fig. 4). Interestingly, even though M2-like bMDMs demonstrated reduced CD11b and CD206 expression, this did not affect the relative inhibition of ADCP capacity that was comparable between M2-like pMDM and bMDMs.

## Discussion

Macrophage growth, differentiation, and function as immune cells have been extensively studied using in vitro methods, which generally utilize human peripheral blood to establish macrophage populations. The choice of tissue, although mainly down to availability and yield of cells, may influence the phenotype and function of differentiated macrophages. In mice, different macrophage populations have already been identified from various tissues, with macrophages derived from bone marrow, spleen, and the peritoneal cavity showing differences in activation in an M0 state as well after M1 and M2-like polarization.^32^ Diverse populations have also been identified in human macrophages with distinct transcriptional and epigenetic profiles evident from different tissues and activation states.^33^ In both mice and human these differences have been attributed to the presence of tissue-resident and monocyte-derived macrophages as well as tissue location.

In the blood there are no circulating macrophages, so all pMDMs located in tissue under inflammatory conditions first differentiate from monocytes produced in the bone marrow.^34^ When analyzed using the adherence method for generating macrophages, a standard protocol for both human peripheral blood- and mouse bone marrow- derived macrophages,^15^^,^^31^ we observed a distinct contamination of stromal cells in the bMDM cultures (Fig. S2), evidenced by an overall reduction in macrophage markers assessed (Figs 1 and 2). Isolating CD14 positive monocytes prior to differentiation removed any stromal cells from the cultures and resulted in similar phenotypes between pMDMs and bMDMs (Figs 3 and 4), with some exceptions including in CD40 expression. Two markers were used to identify an M1-like phenotype including CD38 and CD40, which have been strongly linked to activation and M1 polarization.^25^^,^^35^^,^^36^ CD38 showed similar expression in both pMDM and bMDM cultures, while CD40 had a reduced trend in the bMDMs (Fig. 4); combining these data, along with those in Fig. S3, indicates this difference is negligible and could have been due to donor variations. The level of CD206 expression also showed a nonsignificant decrease in polarized bMDMs compared to pMDMs (Fig. 4), which would likely equalize with more repeats. Multiple macrophage markers have been shown to change depending on the length of stimulation, with studies suggesting the expression of macrophage markers changes over time;^37^ this could also be different between the 2 sources and explain the small differences in expression. Consequently, a combination of markers, as used here, is most appropriate for identifying distinct macrophage populations and is especially important in human macrophages where there can be large sample variability.

The level of activation of macrophages affects their ability to phagocytose targets, with multiple studies showing M1-like macrophages showing a higher level of ADCP compared to M2-like macrophages.^15^^,^^21^^,^^31^ Phagocytosis assays, both Fc independent (Fig. 5) and dependent (Figs 6 and 7), showed that there were no differences in the effector function of M0 macrophages from the 2 sources. The small decrease in M2-like polarization markers in the bMDMs also did not correlate with the level of ADCP for these cells (Fig. 7), which showed similar percentage phagocytosis with the M2-polarized pMDMs. In contrast, M1-like bMDMs demonstrated a reduction in ADCP compared to pMDMs, although this was not significant (Fig. 7). One possibility is this inhibition of activation could be due to the presence of small amounts of lipids in the bMDM culture. Bone marrow has a high adipocyte and fatty tissue content, and although CD14 positive cells were isolated some lipid residue was still visible when the cells were plated and washed. The bMDMs could have taken up those lipids during differentiation, which has previously been shown to inhibit phagocytosis of apoptotic cells and alter the lipid composition of the macrophage plasma membranes.^38^ It should also be noted that the bone marrow was donated by patients having hip replacement surgery, and consequently were likely osteoarthritic or osteoporotic. The limited increase in M1 ADCP compared to M0 could be a result of this diseased source. Another limitation of this study is the lack of investigation at the transcriptomic level, for example, RNA sequencing, which might reveal differences in gene expression between pMDMs and bMDMs before and after polarization. Although changes in mRNA expression do not always correlate with shifts in protein expression, such transcriptional data could be important in understanding any fundamental differences between pMDMs and bMDMs that were not observed here and thereby further inform future investigations utilizing cells from these two sources.

In summary, there were no differences in either phenotype or phagocytic function of M0 macrophages derived from 2 anatomic niches: human PBMC and bone marrow. There were small differences in the phenotype of polarized macrophages, which did result in a nonsignificant reduction of ADCP in M1-like bMDM but did not affect M2-like macrophages. This suggests that these two cell sources could be used interchangeably for monocyte-derived macrophage in vitro assays, although due to increased yield and availability peripheral blood–derived macrophages remain the most suitable source.

## Supplementary Material

vkae032_Supplementary_Data

## Data Availability

The data that support the findings of this study are available in the materials and methods, results and supplemental material of this article.
