# Supplementary material for: Comparison of human macrophages derived from peripheral blood and bone marrow
Source: J Immunol. 2025 Mar 5;214(4):714–25. doi: 10.1093/jimmun/vkae032 (PMC12041772; doi:10.1093/jimmun/vkae032)
Supplement: vkae032_Supplementary_Data [file vkae032_supplementary_data.zip › vkae032_Supplementary_Data/JIMMUN-24-00333-s01.pdf]

Supplementary

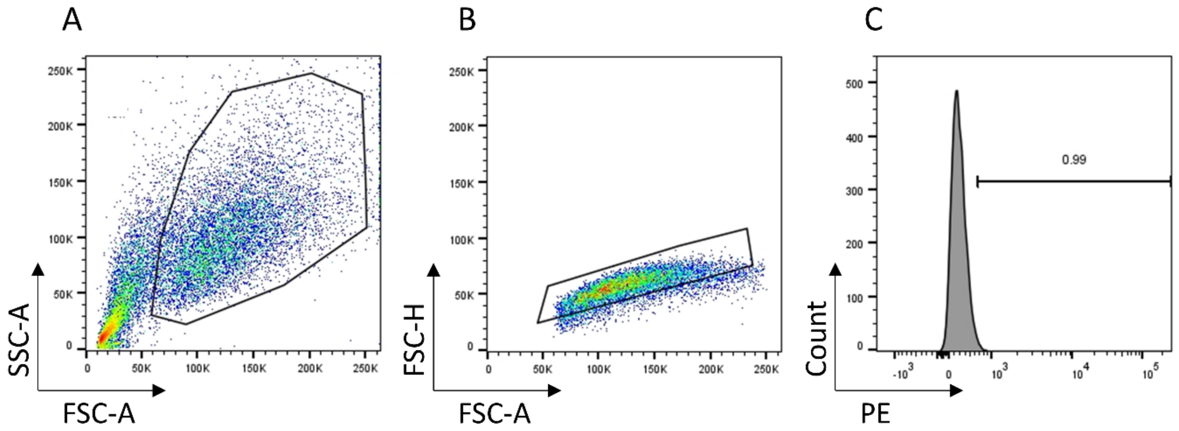

Supplementary Figure 1. Representative flow cytometry gating strategy. *After incubation macrophages were analysed on a BD Canto with data processed using FlowJo (v10). A) Live cells were identified based on size and morphology followed by B) doublet removal. C) Isotype control antibodies were used to identify positive staining.*

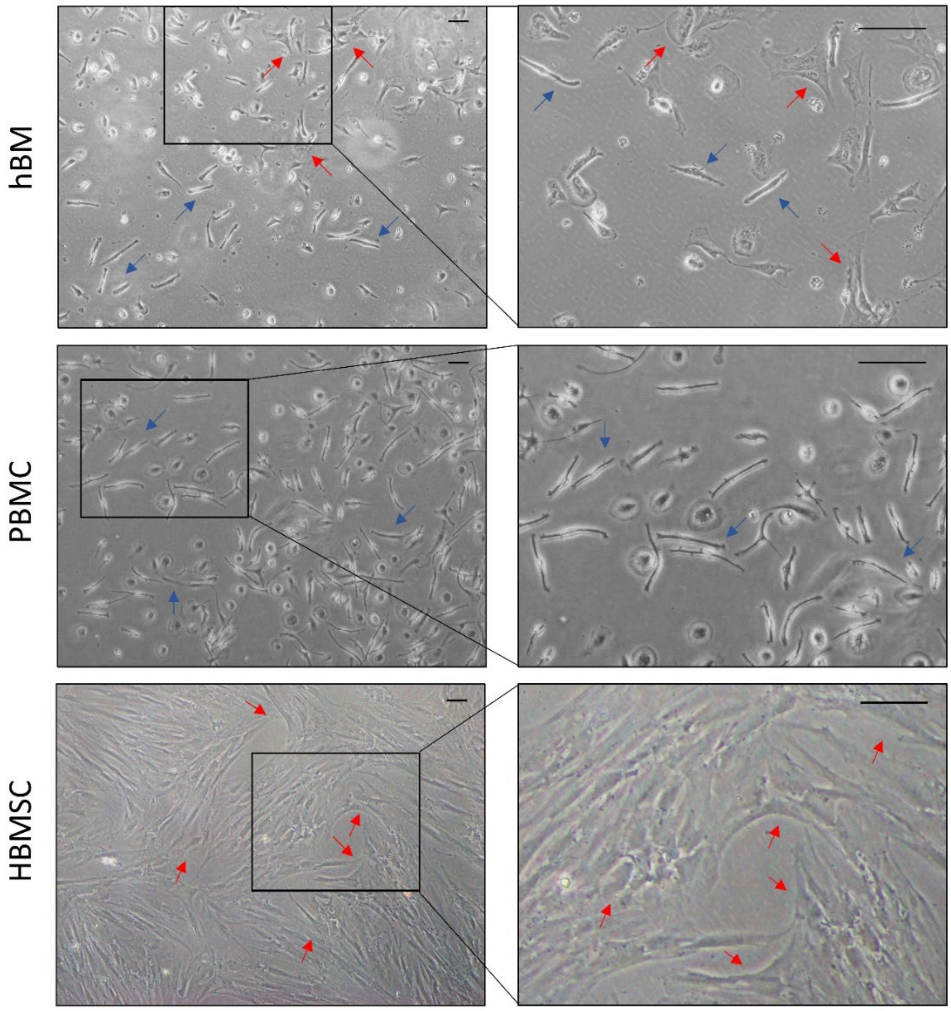

Supplementary Figure 2. Representative images showing contamination of stromal cells into the bone marrow macrophage populations. *A) A heterogenous cell population was isolated from human bone marrow (hBM), and after two hours adherent cells were cultured with M-CSF for 5 days, showing both macrophages and stromal cells. B) Macrophages were differentiated from a heterogeneous cell population of PBMCs, after a two-hour incubation the non-adherent cells were removed, and the remaining cells were cultured with M-CSF for 5 days. C) Stromal cells grown from a heterogenous cell population of human bone marrow and cultured without M-CSF for 14 days. Scale Bar= 100 µm, blue arrows indicate macrophages, red arrows indicate stromal cells.*
